# The neovaginal microbiome of transgender women post-gender reassignment surgery

**DOI:** 10.1186/s40168-020-00804-1

**Published:** 2020-05-05

**Authors:** Kenzie D. Birse, Kateryna Kratzer, Christina Farr Zuend, Sarah Mutch, Laura Noël-Romas, Alana Lamont, Max Abou, Emilia Jalil, Valdiléa Veloso, Beatriz Grinsztejn, Ruth Khalili Friedman, Kristina Broliden, Frideborg Bradley, Vanessa Poliquin, Fan Li, Carolyn Yanavich, Adam Burgener, Grace Aldrovandi

**Affiliations:** 1 National HIV and Retrovirology Labs, JC Wilt Infectious Disease Research Centre, Public Health Agency of Canada, 745 Logan Ave, Winnipeg, MB R3E 3 L5 Canada; 2 Departments of Medical Microbiology and Infectious Disease, University of Manitoba, Winnipeg, MB Canada; 3 Instituto Nacional de Infectologia Evandro Chagas, Rio de Janeiro, Brazil; 4 Department of Medicine Solna, Center for Molecular Medicine, Unit of Infectious Diseases, Karolinska Institutet, Karolinska University Hospital, Stockholm, Sweden; 5 Department of Obstetrics & Gynecology, University of Manitoba, Winnipeg, MB Canada; 6 University of California, Los Angeles, CA USA

**Keywords:** Transgender women, Neovagina, Gender reassignment surgery, Microbiome, Metaproteomics

## Abstract

**Background:**

Gender reassignment surgery is a procedure some transgender women (TW) undergo for gender-affirming purposes. This often includes the construction of a neovagina using existing penile and scrotal tissue and/or a sigmoid colon graft. There are limited data regarding the composition and function of the neovaginal microbiome representing a major gap in knowledge in neovaginal health.

**Results:**

Metaproteomics was performed on secretions collected from the neovaginas (*n* = 5) and rectums (*n* = 7) of TW surgically reassigned via penile inversion/scrotal graft with (*n* = 1) or without (*n* = 4) a sigmoid colon graft extension and compared with secretions from cis vaginas (*n* = 32). We identified 541 unique bacterial proteins from 38 taxa. The most abundant taxa in the neovaginas were *Porphyromonas* (30.2%), *Peptostreptococcus* (9.2%), *Prevotella* (9.0%), *Mobiluncus* (8.0%), and *Jonquetella* (7.2%), while cis vaginas were primarily *Lactobacillus* and *Gardnerella*. Rectal samples were mainly composed of *Prevotella* and *Roseburia*. Neovaginas (median Shannon’s *H* index = 1.33) had higher alpha diversity compared to cis vaginas (Shannon’s *H* = 0.35) (*p* = 7.2E−3, Mann-Whitney *U* test) and were more similar to the non-*Lactobacillus* dominant/polymicrobial cis vaginas based on beta diversity (perMANOVA, *p* = 0.001, *r*^2^ = 0.342). In comparison to cis vaginas, toll-like receptor response, amino acid, and short-chain fatty acid metabolic pathways were increased (*p* < 0.01), while keratinization and cornification proteins were decreased (*p* < 0.001) in the neovaginal proteome.

**Conclusions:**

Penile skin-lined neovaginas have diverse, polymicrobial communities that show similarities in composition to uncircumcised penises and host responses to cis vaginas with bacterial vaginosis (BV) including increased immune activation pathways and decreased epithelial barrier function. Developing a better understanding of microbiome-associated inflammation in the neovaginal environment will be important for improving our knowledge of neovaginal health.

Video Abstract

## Introduction

Transgender is a term used to define people whose gender identity is different from their assigned sex at birth [1]. Many transgender women (TW), defined here as people assigned as male at birth who identify as female, undergo medical interventions such as feminizing hormone therapy and gender reassignment surgery (GRS) for gender affirmation purposes [2]. GRS generally includes neovaginoplasty, during which a neovagina is created through penile inversion, scrotal grafts, sigmoid colon grafts, and/or a combination thereof [3].

There are scarce data about the environment of the neovagina particularly at the molecular and microbial level. For cisgender women (CW), i.e., those assigned as female at birth, it is well understood that optimal vaginal microbiota include *Lactobacillus* species. Meanwhile, non-optimal microbial communities in the cis vagina such as those containing *Gardnerella vaginalis*, *Atopobium*, *Prevotella*, *Mobiluncus*, and other anaerobic species are often higher in diversity and associate with a condition known as bacterial vaginosis (BV). BV or anaerobic dysbiosis has been linked to increased genital tract inflammation and an increased risk of STI acquisition in CW as well as in uncircumcised men [4–7]. However, limited molecular sequencing studies have been conducted on neovaginas for the purposes of defining the microbiome [8, 9]. To address this gap, we chose to investigate the neovaginal microbiome using a metaproteomics technique [10]. The objectives of this study were to map and characterize the microbial composition and function of neovaginal and rectal secretions from TW and compare them with vaginal secretions from CW and to assess relationships between microbial communities measured and host immune pathways.

## Results

### Demographic and clinical characteristics of study participants

We examined rectal and neovaginal secretions from TW (*n* = 9) and compared them against vaginal secretions from CW (*n* = 30). We excluded participants without measurable bacterial protein levels. Five out of the nine (56%) neovaginal samples and seven out of the nine (78%) rectal samples and all cis vaginal samples had measurable bacterial protein levels and remained in the analysis. Age, GRS methods, feminizing hormone therapy, sexually transmitted infections (STI), and sexual behavior data for the TW and CW with measurable neovaginal and vaginal microbiome data are described in Table 1. CW were younger (median age = 31) than TW (median age = 48) (*p* = 0.058, Mann-Whitney *U* test, Table 1). The median time elapsed since last GRS was 9.5 years (range 3.5–34 years).

**Table 1:** Demographic, clinical, and behavioral data of study participants with microbiome data

Variable	Variable category	Transgender women—neovaginal samples (*n* = 5)^a^	Transgender women—rectal samples (*n* = 7)^a^	Cisgender women—vaginal samples (*n* = 30)^b^
Age (median, range)		48 (28–57)	48 (35–57)	31 (19–55)
Time since first neovaginoplasty (median, range)	Years	9.5 (3.7–35.7)	10.9 (5.1–35.7)	NA
Time since most recent neovaginoplasty (median, range)	Years	9.5 (3.5–34)	9.5 (1.5–34)	NA
Surgery method—all techniques (*n*, %)	Penile inversion, scrotal cutaneous graft	4 (80)	4 (57)	NA
	Penile inversion, scrotal cutaneous graft with sigmoid colon extension	1 (20)	3 (43)	
Total number of surgeries (median, range)^c^		1 (1–4)	2 (1–6)	NA
Vaginal complaints (*n*, %)^d^		2 (40)	3 (43)	13 (43)
Number who practice vaginal washing (*n*, %)		1 (20)	1 (20)	1 (3.3)
Hormone therapy (*n*, %)^e^	Estrogen therapy/hormonal contraception	3 (60)	5 (71)	11 (37)
	Not currently on therapy/hormonal contraception	1 (20)	1 (14)	16 (53)
	Unknown	1 (20)	1 (14)	3 (10)
STI (*n*, %) ^f^	Hepatitis B	4 (80)	5 (71)	Data not collected
	Hepatitis C	0 (0)	0 (0)	Data not collected
	Active syphilis	0 (0)	1 (14)	Data not collected
	Anal chlamydia	0 (0)	2 (29)	Data not collected
	Vaginal chlamydia	0 (0)	1 (14)	1 (3.3)
	Anal gonorrhea	1 (20)	0 (0)	Data not collected
	Vaginal gonorrhea	0 (0)	0 (0)	0
	HIV	0 (0)	0 (0)	2 (6.7)
Sexually active in 30 days prior to sample collection (*n*, %)^g^	Reported receptive anal sex	1 (20)	3 (43)	NA
	Reported receptive neovaginal sex	2 (40)	4 (57)	NA
	Reported cis vaginal sex	NA	NA	19 (63)
Condom use in the last 30 days (*n*, %) ^h^		0 (0)	0 (0)	7 (23)
Number of sexual partners in the last 30 days (median, range)		1 (0–1)	1 (0–1)	Data not collected
Applied a chemical substance (*n*, %)^i^		2 (40)	2 (29)	Data not collected
Used sex toy in vagina (*n*, %)		2 (40)	1 (14)	Data not collected

^a^Nine transgender women (TW) had both neovaginal and rectal samples collected for metaproteomics, but only 5 neovaginal samples and 7 rectal samples had bacterial proteins detected

^b^A total of 32 samples from 30 cisgender women (CW) were collected. Two samples were longitudinal from the same Swedish participants

^c^Reasons for re-operation included stenosis, procedural complications, dissatisfaction with external appearance, anorgasmia, and sexual mismatch

^d^Vaginal complaints included spontaneous pain, pain during intercourse, spontaneous bleeding, fetid odor and/or vaginal depth amongst TW, and vaginal discomfort, discharge, itching, pain, bleeding, and odor amongst CW

^e^CW reported using either oral contraceptive pills, injectable contraception, vaginal ring contraception, or a hormonal intrauterine device

^f^All STIs were tested for at the time of sample collection. Hepatitis B seropositivity represented past exposure

^g^Includes instances of neovaginal and/or anal intercourse for TW and penetrative vaginal intercourse for CW

^h^Indicates condom use in any sexual activities (anal and neovaginal intercourse included) in the 30 days prior to sample collection

^i^Chemical substances included lubricants, soap, and/or douche

### Neovaginal secretions contain diverse communities of anaerobic bacteria

**Fig. 1:**
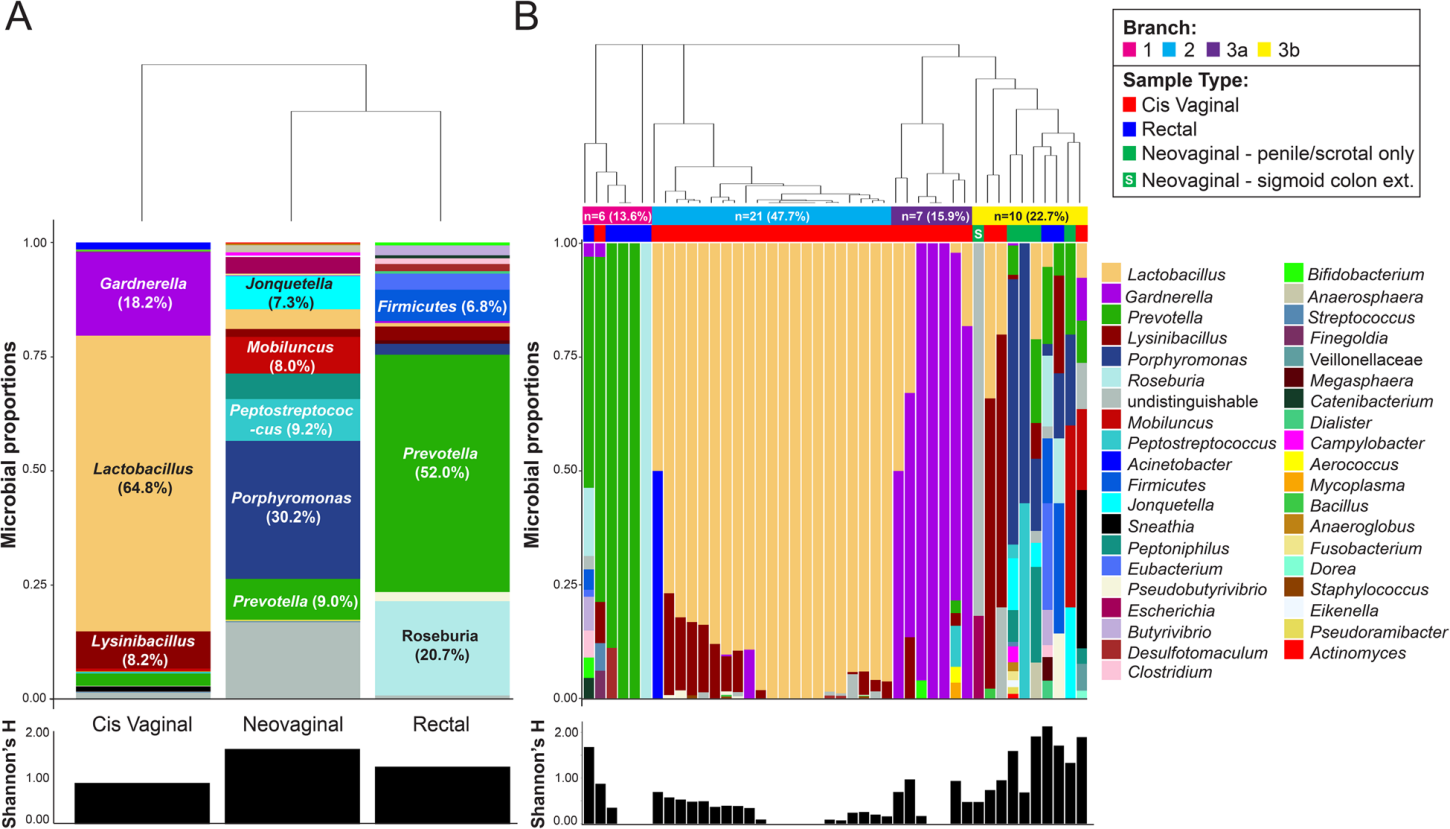
Microbial profiles determined by mass spectrometry reveal distinct microbial community structures in the neovaginal, rectal, and vaginal compartments from transgender and cisgender women, respectively. **a** Unsupervised, hierarchical clustering performed on the averaged proportional abundance of the bacterial taxa detected suggests the polymicrobial, neovaginal profile on average is more similar to the rectal profile compared to the cis vaginal profile which is dominated by *Lactobacillus* and *Gardnerella*. **b** Hierarchical clustering of the individual profiles reveals that neovaginal profiles specifically cluster together as well as with other diverse, species-rich cis vaginal and rectal profiles (branch 3b). All of which are significantly more diverse (Shannon *H* Index, *p* = 5.0E−4, Kruskal-Wallis) than cis vaginal and rectal profiles found in branches 1, 2, and 3a

Mass spectrometry analysis identified a total of 541 unique bacterial proteins from 38 genera belonging to 7 different phyla: *Actinobacteria*, *Bacteroidetes*, *Firmicutes*, *Fusobacteria*, *Proteobacteria*, *Synergistetes*, and *Tenericutes*. We identified on average 8 species per neovaginal sample (range 2–17), 6 species per rectal sample (range 1–18), and 5 species per cis vaginal sample (range 1–14). On average, the most prevalent taxa by protein abundance in the neovaginal samples were *Porphyromonas* (30.2%), *Peptostreptococcus* (9.2%), *Prevotella* (9.0%), and *Mobiluncus* (8.0%), with a large proportion undistinguishable (16.9%) (median bacterial spectral count = 63, range = 14.5–177.9). In rectal secretions, the most abundant bacterial taxa were *Prevotella* (52.0%), *Roseburia* (20.7%), *Firmicutes* (6.8%), *Eubacterium* (3.6%), and *Lysinibacillus* (3.1%) (median bacterial spectral count = 12.13, range = 7.5–435.7). In cis vaginal secretions, the most abundant taxa were *Lactobacillus* (64.8%), *Gardnerella* (18.2%), *Lysinibacillus* (8.2%), and *Prevotella* (2.7%) (median bacterial spectral count = 113.2, range = 4.1–456.3) (Fig. 1a). 16S rRNA gene analysis also detected many of these major taxa identified via metaproteomic analysis as well as many other taxa in neovaginal, rectal, and cis vaginal samples, respectively (Supplemental Figures 1, 2 and 3). Total bacterial signal did not significantly differ based on sample type (*p* = 0.34, Kruskal-Wallis).

**Fig. 2:**
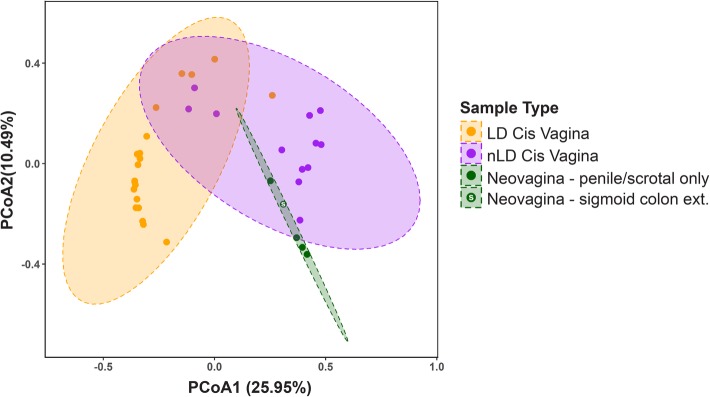
Principal coordinate analysis demonstrates that neovaginal bacterial composition is more similar to that of non-*Lactobacillus*-dominant (nLD)/polymicrobial than *Lactobacillus*-dominant (LD) cis vaginas based on Bray-Curtis dissimilarity distances

Hierarchical clustering of individual profiles revealed 3 main branches based on bacterial protein composition (Fig. 1b). Branch 1 included 5 rectal samples, and 1 cis vaginal sample dominated by *Prevotella* or *Roseburia*. Branch 2 and 3a were composed entirely of cis vaginal samples who were dominated by *Lactobacillus* or *Gardnerella*, respectively. Branch 3b included all neovaginal samples (*n* = 5) as well as 3 cis vaginal and 2 rectal samples. Branch 3b had significantly higher alpha diversity based on Shannon’s *H* diversity than branch 1, 2, and 3A (2.77, 5.27, and 2.9 fold changes, respectively; Shannon’s *H* index, *p* = 0.0005, Kruskal-Wallis). Neovaginal samples had higher alpha diversity (Shannon *H* index median = 1.33) than cis vaginal samples (Shannon *H* median = 0.35) (*p* = 0.0072, Mann-Whitney *U* test) when examined separately. Indeed, neovaginas grouped more closely with non-*Lactobacillus* dominant/polymicrobial (< 50% *Lactobacillus* proteins contribute to the microbial profile) than cis vaginas when Bray-Curtis dissimilarity distances were examined (Fig. 2). Variation in bacterial community composition between individuals can be attributed to sample type (*p* = 0.001, *r*^2^ = 0.13, perMANOVA) and *Lactobacillus* levels (*p* = 0.001, *r*^2^ = 0.21 perMANOVA). 16S rRNA gene profiling also revealed similarities between the neovaginal and non-*Lactobacillus* dominant/polymicrobial cis vaginal bacterial communities (Supplemental Figures 1 and 2) highlighted by the increased abundance of *Prevotella* and increased overall diversity.

Proteins annotated to *Jonquetella anthropi*, the only bacteria identified belonging to the phylum *Synergistetes*, were uniquely identified in 60% of neovaginal samples. 16S rRNA genes belonging to family *Synergistaceae* were detected in 80% the neovaginal samples. Other unique taxa identified in at least one neovaginal sample included various *Proteobacteria* (*Escherichia*, *Campylobacter*, *Eikenella*), *Firmicutes* (*Anaerosphaera*, *Anaeroglobus*, *Pseudoramibacter*), *Fusobacteria* (*Fusobacterium*), and *Actinobacteria* (*Actinomyces*). Interestingly, the one neovaginal sample that had a sigmoid colon graft for neovaginal extension purposes had a microbiome that appeared more gut-like such that *Bacteroidaceae* and *Enterobacteriaceae* were the main taxa detected via 16S rRNA gene sequencing. This was partially confirmed via metaproteomics as there were *Escherichia* proteins detected as well (Supplemental Figure 1). This participant’s matching rectal sample (R6) was also composed of elevated levels of *Bacteroidaceae* (41%) as measured by 16S rRNA gene sequencing (Supplemental Figure 3).

A subset of the samples (*n* = 4) included in this study had matched neovaginal and rectal secretions collected (Supplemental Figure 4A). Principal coordinate analysis highlighted the differences in microbial composition based on bacterial proteins that exist between each of the rectal-neovaginal sample pairs based on beta diversity (Bray-Curtis distances) (Supplemental Figure 4B). Variation in bacterial community composition between rectal and neovaginal samples can be attributed to sample type (*p* = 0.014, *r*^2^ = 0.24, perMANOVA) and *Roseburia* levels (*p* = 0.02, *r*^2^ = 0.20, perMANOVA).

### Microbial functional differences exist between neovaginal and cis vaginal samples

Of the 541 bacterial proteins identified, 377 (70%) were successfully assigned functions from the KEGG Pathway database. The top five most abundant broad, B-level functions in neovaginal samples included energy metabolism (29.8%), carbohydrate metabolism (23.2%), amino acid metabolism (17.8%), metabolism of cofactors and vitamins (9.3%), and signal transduction (7.6%). The top B-level functions in cis vaginal samples were carbohydrate metabolism (37.5%), energy metabolism (17.5%), signal transduction (8.2%), metabolism of cofactors and vitamins (6.1%), and membrane transport (5.1%) (Supplemental Figure 5). Upon further evaluation of more specific functional categories (KEGG ko level), vitamin B6 metabolism via phosphoserine aminotransferase from *Poryphromonas* and various forms of amino acid and fatty acid metabolism were uniquely associated with neovaginal samples (Supplemental Table 1).

## The neovagina associates with increased immune activation and decreased barrier function pathways

**Fig. 3:**
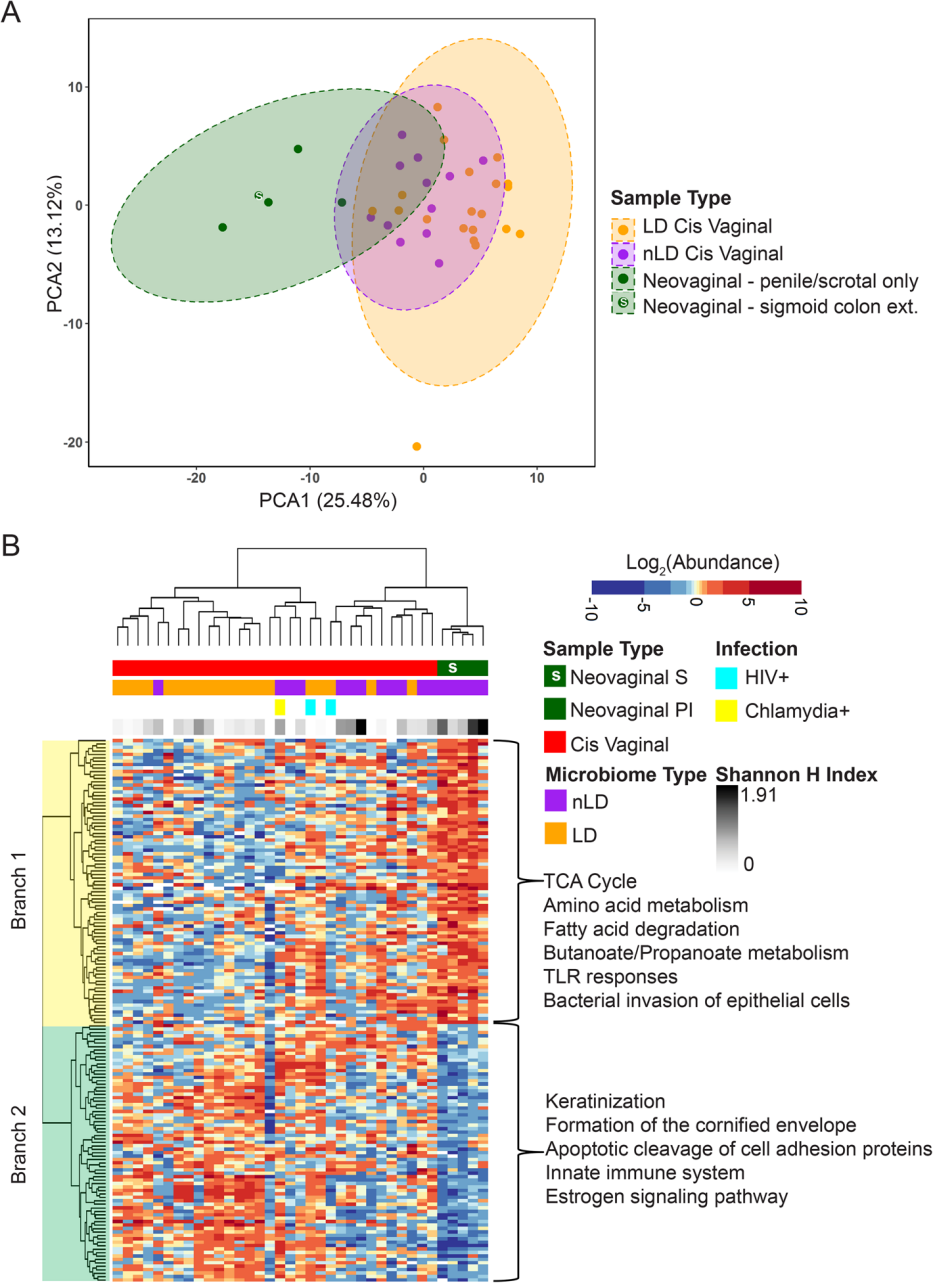
Increased immune activation and decreased keratinization and barrier pathways are associated with the neovagina. **a** Neovaginal samples have an overall unique host proteome signature compared to cis vaginal samples based on variation decomposition via principal coordinate analysis. **b** Hierarchical clustering of human proteins differentially abundant between neovaginas and cis vaginas (*p* < 0.05, Mann-Whitney *U* test). Neovaginas had increased signatures of immune activation and bacterial invasion and decreased barrier function and estrogen signaling pathways (*q* < 0.05, ConsensusPathDB). (LD = *Lactobacillus* dominant, nLD = non-*Lactobacillus* dominant, neovaginal S = sigmoid colon graft extension, neovaginal PI = penile inversion/scrotal graft only)

To explore host immunity differences between neovaginas and cis vaginas, we performed differential protein expression analysis; 158 (15.1%) proteins were significantly different between neovaginas and cis vaginas (*p* < 0.05, Mann-Whitney *U* test, Supplemental Table 2). Of those 158, 68 met the 80% power restriction based on an effect size of 2.7-fold difference. Principal component analysis and hierarchical clustering highlighted how the abundance of these proteins differs between neovaginal and cis vaginal samples (Fig. 3).

Within the hierarchical clustering analysis, two major branches were observed (Fig. 3b). Branch 1 included proteins with known functions in the citrate cycle (8 proteins, *q* = 8.8E10), various forms amino acid metabolism (arginine and proline (6 proteins, *q* = 1.84E−5); valine, leucine and isoleucine (5 proteins, *q* = 1.4E−4), histidine (4 proteins, *q* = 1.4E−4); alanine, aspartate, glutamate (4 proteins, 4.73E−4); tyrosine (*n* = 4, 3.99E-4); phenylalanine (3 proteins, *q* = 4.82E−4); and lysine degradation (3 proteins, *q* = 1.78E-3)), glyoxylate metabolism (4 proteins, *q* = 3.16E−4), Fc gamma R-mediated phagocytosis (5 proteins, *q* = 1.05E−3), toll-like receptor (TLR) responses (TLR JNK (3 proteins, *q* = 1.73E−2), TLR p38 (3 proteins, *q* = 1.74E−2), TLR NFKB (3 proteins, *q* = 1.92E-2), bacterial invasion of epithelial cells (3 proteins, *q* = 2.13E−2), and short chain fatty acid metabolism (butanoate (2 proteins, *q* = 1.55E02), proponaote (2 proteins, *q* = 2.22E−2), all of which were increased in the neovagina (Supplemental Table 3). There were also decreased levels of proteins associated with keratinization (19 proteins, *q* = 1.22E−21), cornified envelope formation processes (10 proteins, *q* = 7.99E−11), apoptotic cleavage of cell adhesion proteins (*n* = 3, *q* = 4.20E−4), the estrogen signaling pathway (4 proteins, *q* = 2.18E−2), and the innate immune system (15 proteins, *q* = 2.44E−3) including various antimicrobial/complement (CAMP, CFH, LCN2, CRISP3, C6) and cell redox homeostasis proteins (TXN, QSOX1) in the neovagina (Supplemental Table 4). Many of these same functions were correlated with alpha diversity (Shannon’s *H* index) (Supplemental Table 5, 6 and 7).

Furthermore, we performed protein set enrichment analysis comparing our data set against pre-defined protein sets from cervical immune cells. The protein set that was the most enriched and overlapped with the proteins found to decrease in the neovaginal compartment relative to the cis vaginal compartment were from CD4+CD38+HLADR+ T cells (normalized enrichment score = − 2.01, FDR *q* value = 1.16E−3) (Supplemental Figure 6, Supplemental Table 8).

## Discussion

Anaerobic bacterial species dominated the neovaginal microbiome. The neovaginal microbial profiles identified in this study overlap with what has been seen in previous penile skin-lined neovaginal studies as well as uncircumcised penile studies including elevated levels of *Prevotella*, *Porphyromonas*, and *Peptoniphilus* (*Clostridiales Family XI*) [7, 8, 11–15]. Indeed, the bacterial composition of penile skin-lined neovaginas resembled those of uncircumcised penises with penile community state types (CST) known to be abundant with BV-associated bacteria [14].

Despite the great deal of consistency of taxa observed in our study and others, several unique taxa were also identified: *Eikenella*, *Anaeroglobus*, *Anaerosphaera*, and *Pseudoramibacter*. Bacteria identified in the neovagina may represent bacteria that were seeded by unique routes of transmission. For instance, *Eikenella corrodens* is a commensal bacteria found in the mouth. Oral-genital contact has been suggested as a possible route of transmission of these bacteria to the genital tract [16]. *Anaeroglobus geminatusa*, *Pseudoramibacter alactolyticus*, *Campylobacter ureolyticus*, *Fusobacterium nucleatum*, and *Actinomyces* have been described as putative pathogens also found in the oral cavity associated with periodontitis and endodontic infections [17–21]. The presence of oral bacteria in the neovaginal compartment could suggest oral-genital bacterial transmission. *Jonquetella anthropi* has been detected on the scrotum and penis and has also been described as an opportunistic pathogen associated with soft tissue infections [7, 22, 23]. Taxa belonging to the phylum *Synergistetes* have been detected from healthy cis vaginas, although we did not see any detected in the cis vaginal samples analyzed in our study [22]. Due to the detection of *J. anthropi* from scrotal/penile samples in other studies, and the fact that the penile inversion/scrotal graft surgical method was the main surgical method used on the TW in this study, there may have been carry over or seeding of these microbiota from the original penis and/or scrotum into the neovagina. Indeed, 3 out of the 4 participants and 4 out of 4 participants who had penile inversion/scrotal graft neovaginoplasty surgery method conducted without a sigmoid colon graft had detectable *J. anthropi* proteins and had measurable *Synergistaceae* 16S rRNA gene sequences, respectively, in their neovaginal samples. Furthermore, the one neovaginal sample that had a sigmoid colon graft in addition to the penile inversion/scrotal graft surgical method had a microbiome that appeared more gut-like as *Bacteroidaceae* and *Enterobacteriaceae* were the main taxa dominating its microbial profile. This finding provides further evidence that the organs used to generate and/or modify the neovagina represent major sources of bacterial transmission or origination that may contribute to the neovaginal microbiome.

Some studies suggest that the vaginal compartment is seeded by bacteria found in the rectal compartment [9]. We found very little bacterial protein-based compositional similarity between matching neovaginal and rectal profiles based on bacterial proteins measured, although we were underpowered to properly evaluate this comparison. The rectal microbial profiles observed in our study were similar to those of other studies particularly those that examined rectal/anal microbiomes of CW as well as men who have sex with men where *Prevotella* and *Bacteroides* were most abundant [24–26].

Various taxa that are associated with BV in CW were detected in the neovagina, including elevated levels of *Prevotella*, *Mobiluncus*, *Porphyromonas*, and *Peptostreptococcus* [27]. Neovaginas also had similar host responses to cis vaginas with BV such that we observed increased immune activation signatures including increased amino acid metabolism, short-chain fatty acid metabolism, TLR responses, and bacterial invasion/phagocytosis, as well as decreased signatures of barrier and innate immune function [28–30]. Decreased levels of particular antimicrobial and/or defense proteins such as cathelicidin (CAMP) and lipocalin-2 (LCN2) may hinder appropriate immune responses to non-optimal bacteria [31, 32]. Decreases in LCN2 may lead to increased bacteria-driven inflammation as this protein has been shown to limit inflammation by restricting bacterial access to iron [33]. Increased amino acid metabolism, particularly the degradation of isoleucine, leucine, and valine, has been linked with antimicrobial protein expression including beta-defensins and mucosal barrier function [34, 35]. Therefore, a lack of these amino acids or amino acid starvation could impair barrier function. This has also been shown to trigger inflammation and T helper 17 cell responses [36, 37]. Furthermore, bacterial vitamin B6 metabolism, a bacterial function uniquely associated with neovaginas in our study, may also be linked to increased host inflammation and hindered immune responses as vitamin B6 levels have been shown to be inversely correlated with various pro-inflammatory markers. A deficiency in vitamin B6 is associated with reduced lymphocyte proliferation, T cell-mediated cytotoxicity, and antibody production [38, 39]. We also found that neovaginal host signatures overlapped with signatures associated with elevated, activated CD4+ T cell levels in the female genital tract providing further evidence of increased immune activation signatures are being detected within the neovagina. Overall, these data suggest that neovaginas are similar to polymicrobial or BV-like cis vaginas based on the bacterial composition, bacterial function, and the corresponding host immune activation and barrier dysfunction profiles.

Neovaginas generated from penile and scrotal skin, which are known to express estrogen receptors, may also have an intrinsic pre-disposition to decreased barrier protein expression due to low estrogen levels relative to the cis vagina [40, 41]. Indeed, we found estrogen-regulated keratins at lower levels in the neovagina as well as a number of cornified envelope proteins. The cornified envelope, as well as the corneodesmosomes found within, is critical to maintaining barrier integrity in tissues that experience mechanical stress such as the neovaginal or vaginal skin [42], and if these are weakened, neovaginas may be more likely to experience tears and/or damage and have limitations to their wound healing potential [43–45]. It is also well understood that estrogen promotes keratinization and barrier integrity in the vagina of animal models as well as in the inner foreskin in humans, and a lack of estrogen or its receptors results in a loss of the cornified layer [46–48]. Of the 5 neovaginal samples included in our analysis, three reported taking estrogen transdermally; therefore, it is possible our observations could be related to a lack of intrinsic and/or pharmaceutically delivered estrogen.

While there was considerable overlap, we observed different bacterial information from 16S rRNA gene sequencing and mass spectrometry-based proteomics. This is not unexpected as these two methodologies measure different components of the microbiome. Proteomic data better reflects the metabolic state of a bacterium which may be dependent upon many factors including the growth state, nutrient availability, and composition of the neighboring microbiota [49–51]. 16S rRNA gene data provides more sensitive data on bacterial composition, but does not provide information on bacterial activity. Therefore, it is not unexpected to observe differences in the proteomic and genomic data in this study.

Limitations exist in this study including its small sample size and post hoc study design. Four of the TW included in this study had the penile inversion/scrotal graft surgical method used for their neovaginoplasties, and one TW had the penile inversion/scrotal graft method with a sigmoid colon graft extension. Future studies with larger sample sizes will be required to better compare the impact of surgical method (i.e., penile inversion versus sigmoid colon grafts) on the microbiome. Further to this, future studies should include uncircumsised penile samples as a comparator to penile skin-lined neovaginal samples. Another limitation in our study was that the CW were not from the same geographic location as the TW and therefore may introduce underlying variation between study groups. There are a few methodological limitations in our study that are important to note. Shotgun proteomics is less sensitive than other sequencing methods such as 16S rRNA sequencing and metagenomics, and therefore, fewer bacterial species were detected by metaproteomics. The 16S rRNA gene sequencing methods used on CW and TW samples were different (V3–V4 vs V4 regions, respectively), as the CW data were originally generated for an independent study. Utilizing different regions of the 16S rRNA gene can impact which bacteria are preferentially amplified, and this represents a limitation to this study. Furthermore, the CW and TW 16S rRNA gene data were each processed using different bioinformatics pipelines, which could also introduce biases in each data set. Nevertheless, we do not expect these to be major contributing factors to the observations in this study. Despite these limitations, this is the first study to evaluate the microbiome of the neovagina using a metaproteomics technique where both bacterial composition and function can be described and related to host responses.

## Conclusions

This study identified unique bacteria in the neovaginal compartment which may have been transmitted via the oral-genital route and/or may represent bacteria originally associated with the organs used to generate and/or modify the neovagina. This study corroborates previous neovaginal studies identifying neovaginas with diverse, polymicrobial communities that elicit similar host responses to cis vaginas with BV. Increased immune activation and reduced barrier protein signatures detected within the neovaginal compartment, whether caused by the bacteria present or an intrinsic lack or insufficient level of pharmaceutically delivered estrogen, are important findings that increase our understanding of the physiology of the neovagina.

## Methods

### Study populations and ethics statement

This is a cross-sectional study that evaluated Brazilian, TW recruited at the LaPClin-AIDS Clinical Research Laboratory of the National Institute of Infectious Diseases Evandro Chagas (INI), at Oswaldo Cruz Foundation (FIOCRUZ) in Rio de Janeiro, Brazil, and CW from Canada and Sweden. All TW were over the age of 18 and were tested for STIs including HIV, chlamydia, gonorrhea, syphilis, and hepatitis B and C. CW were also over the age of 18, not pregnant, and were tested for HIV, chlamydia, and gonorrhea. Swedish participants included in our CW control group were from a low-risk cohort not taking any form of hormonal contraception. Canadian participants included in our CW control group were from a higher-risk cohort of women experiencing negative reproductive health outcomes including vaginal symptoms and/or HIV/STI infections. Women whose samples had no measurable bacterial protein levels were excluded. The study was approved by the Research Ethics Committee at the INI-FIOCRUZ, the Research Ethics Board of the University of Manitoba, and the Stockholm Regional Ethics Board. Written informed consent was obtained from all study participants.

### Sample collection

Secretions were collected by swabbing the neovaginal and rectal compartments from TW who had undergone GRS. Cervicovaginal secretions were collected from CW via swab or cervicovaginal lavage*.* Secretions from TW were placed in cryotubes of 500 μL Allprotect (Qiagen, Valencia, CA) and then frozen at − 80 °C. Secretions from CW were frozen at − 80 °C shortly after collection.

### Sample preparation for mass spectrometry

Swabs were centrifuged to remove excess Allprotect. Swabs were eluted with phosphate-buffered saline (pH 7.0) at 4 °C. Eluates were centrifuged to remove cellular debris and stored at − 80 °C. Equal volumes and/or concentrations of each sample were digested with trypsin and analyzed by tandem mass spectrometry as described by Birse et al. [64]. Briefly, samples were denatured with urea, reduced with diothiothreitol, alkylated with iodoacetamide, and digested with trypsin into peptides. Peptides were cleaned of salt and detergents by reverse-phase liquid chromatography (LC) using a step-function gradient. Cleaned peptides were quantified using LavaPep’s Fluorescent Peptide and Protein Quantification Kit (Gel Company, CA, USA) according to the manufacturer’s protocol.

### Mass spectrometry analysis

One microgram of peptide per sample was re-suspended in 2% acetonitrile, 0.1% formic acid, and injected into a nano-flow LC system (Easy nLC, Thermo Fisher, MA, USA) connected inline to a Q Exactive Quadrupole Orbitrap mass spectrometer (Thermo Fisher, MA, USA). The Q Exactive mass spectrometer (MS) used the following method: a 50-cm long, 2.0-μm particle-sized Easy-Spray C-18 column (Thermo Fisher, MA, USA) was used for peptide separation. The elution gradient was from 98% buffer A to 30% buffer B in 200 min at a constant flow rate of 200 nL/min. MS spectra were acquired on the Orbitrap analyzer at 70,000 resolution at 200 m/z. After each MS spectrum and automatic selection, the 15 most intense precursor ions were selected from fragmentation by high collision dissociation, at 28% normalized collision energy, and were acquired in the Orbitrap analyzer at 17,500 resolution at 200 m/z.

Bacterial peptide identity searching was performed using Mascot (v2.4; Matrix Science, Boston, MA). Data were searched against a manually curated TrEMBL (Translated European Molecular Biology Laboratory) database containing the major genera identified in an initial search against all TrEMBL bacterial proteins. The curated database contained 57 different bacterial taxa (Supplemental Table 9) and the database from *Homo sapiens* to rule out potential homologs. Human peptide identity searching was performed with Mascot v2.4.0 (Matrix Science) against the human SwissProt database. A decoy database was included to determine the false discovery rate. Search results for both human and bacteria were imported into scaffold separately to validate the protein identifications, using the following criteria: ≤ 0.1% false discovery rate (FDR) for peptide identification, ≤ 1% FDR for protein identification, and at least 2 unique peptides identified per protein. Microbial abundance was calculated by summing normalized total spectral counts for all proteins associated with each genus. Host proteome results were imported into Progenesis LC-Mass Spectrometry software to perform label-free differential protein expression analysis based on MS peak intensities. Feature detection, normalization, and quantification were all performed using default settings from the software.

### Functional microbiome analysis

Functional microbiome analysis was performed using KEGG (Kyoto Encyclopedia of Genes and Genomes) ontology assignment through the GhostKOALA (KEGG Orthology And Links Annotation) portal. Pathway maps were reconstructed using observed proteins and manually curated to remove 7 categories associated with organism-level functions (aging, cardiovascular diseases, endocrine and metabolic diseases, endocrine system, immune system, nervous system, neurodegenerative diseases), protein not found in the database, and 2 general “overview” categories to eliminate redundancy (global overview and maps, cancers: overview). Cumulative functional abundance for each category was calculated by summing abundances of all associated protein spectral counts, and proteins belonging to multiple categories contributed to each of those associated.

### Protein set enrichment analysis

Pre-ranked log_2_ mean fold change difference values (neovagina vs cis vagina) were uploaded to the Broad Institute’s gene set enrichment tool (http://www.broadinstitute.org/gsea), and compared against a cervical immune cell library with phenotypes defined via flow cytometry, and high vs low levels separated via median cell count. The cervical immune cells were phenotyped using the following antibodies: CD19-APC, CD56-PE-Cy7, CCR5-FITC, CD38-BUV737, CD184-BV650, CD4-APC-H7, CD3-v500, CD8-BV605, HLA-DR-PE-CF594, CCR6-BV711, CD49d-PE-Cy5, beta-7 integrin-BV421, CD14-PerCP-Cy5.5, CD16-Alexa700, CD15-BUV395, and fixable viability stain 570 (all antibodies obtained from BD Biosciences). Samples were fixed in 1% paraformaldehyde prior to acquisition on an LSR II (BD Biosciences). Human peripheral blood mononuclear cells were used for staining controls and fluorescence minus one controls. Data was analyzed using FlowJo v10 (Treestar).

The protein set size parameters were set between 15 and 500 proteins associated. Protein sets with an FDR *q* value below 0.05 were included in our analysis. For protein sets with overlapping associations with our data set, only those with a normalized enrichment score greater than 2 are shown. We defined normalized enrichment scores (NES) greater than an absolute value of 2.0 as high scoring, NES > |1.5| as medium scoring, and NES < 1.5 as weak scoring. Enrichment scores are assigned based on how similar each protein is ranked between the two data sets. The greater the overlap and consistent ranking of proteins between our data set and the pre-defined data set, the higher the enrichment score. The rank metric score is the score used to position the gene in the ranked list and represents each protein’s correlation with your phenotype of interest (i.e., neovaginal vs cis vagina).

### 16S rRNA gene analysis

#### Canadian cis vaginal 16S rRNA gene analysis

DNA extraction from 75 μL of cervicovaginal lavage (CVL) was carried out in triplicate for each biological sample using the DNeasy Blood and Tissue kit (QIAGEN, Inc., Toronto, ON) with modifications to increase lysis [29]. These modifications were to mix 10 μL lysozyme (100 mg/mL stock), 2.5 μL mutanolysin (25 U/μL stock), and 90 μL TES buffer (25 mM Tris-HCl (pH 8.0), 10 mM EDTA, 10% (w/v) sucrose) with each sample and incubate at 37 °C for 30 min. Next, 600 μL of lysis buffer (100 mM Tris, 100 mM EDTA, 10 mM NaCl, 1% SDS, pH 8.0) was added, followed by a 15-min incubation at room temperature. Then, 25 μL proteinase K and 200 μL buffer AL (Qiagen) were added, and samples were incubated at 56 °C for 30 min. After this step, the manufacturer’s protocol (Qiagen) was followed for the remainder of extraction. Mock community and water controls were processed alongside samples. DNA was amplified using 341F and 785R primers targeting the V3–V4 region of the 16S rRNA gene [52], with overhang adaptor sequences added for sequencing [53]. Full primer sequences including this overhang were 5′-TCGTCGGCAGCGTCAGATGTGTATAAGAGACAGCCTACGGGNGGCWGCAG-3′ for the forward primer, and 5′-GTCTCGTGGGCTCGGAGATGTGTATAAGAGACAGGACTACHVGGGTATCTAATCC-3′ for the reverse primer. The PCR reaction was completed in a total volume of 25 μL, with each reaction containing 1 μL of template DNA, 16.25 μL UltraPure™ DNase/RNase-free distilled water (Thermo Fisher, Mississauga, ON), 2.5 μL PCR Gold Buffer (10×), 2.5 μL MgCl_2_ (25 mM), 0.5 μL dNTPs (10 mM), 1 μL forward primer (0.5 μM), 1 μL reverse primer (0.5 μM), and 0.25 μL AmpliTaq Gold™ Taq DNA polymerase (5 U/μL) (Thermo Fisher, Mississauga, ON). Samples were denatured at 95 °C for 3 min then underwent 35 cycles of 95 °C for 30 s, 55 °C for 30 s, and 72 °C for 30 s, followed by a final extension at 72 °C for 5 min.

PCR products were purified using Ampure XP beads (Beckman Coulter, Mississauga, ON) and run on QIAXcel Advanced Instrument (QIAGEN, Inc., Toronto, ON) to check amplicon purity and band size. All samples were amplified to add sequencing adaptors in a second PCR, using Nextera XT Index Kit v2 Set A and Set D (Illumina Inc., San Diego, CA, USA). This PCR reaction was completed in a total volume of 50 μL and had 8 cycles of 95 °C for 30 s, 55 °C for 30 s, 72 °C for 30 s, followed by extension at 72 °C for 5 min. PCR products were again purified using Ampure XP beads and run on QIAXcel Advanced Instrument (QIAGEN, Inc., Toronto, ON) to check amplicon purity and band size. DNA concentration in nanograms per microliter was quantified using Qubit 2.0 fluorometer (Life Technologies, Inc., Burlington, ON), after which concentration of all samples was normalized to 4 nM. Samples were prepared for MiSeq following manufacturer’s protocol (Illumina Inc., San Diego, CA, USA). Final pooled DNA was diluted to 8 pM, and a spike-in of 10% PhiX was run with pooled samples. Experiment was run on Illumina MiSeq using 500 cycle v2 PE reagents, resulting in 2 × 250 bp paired-end reads.

Reads were analyzed using mothur v1.39.5 [54] and following outline of the MiSeq SOP [55]. Briefly, forward and reverse reads for each sample were joined into contigs and the primer sequences were trimmed. The tertiary quartile of contig length was found to be 427 bp, and therefore, any contigs over 427 bp in length were discarded. A custom version of the 16S rDNA SILVA reference alignment (v132) [56] was made specific to the V3–V4 region of the 16S rRNA gene, and contigs were aligned to this reference. Any sequences that did not align were discarded. Sequences with up to 2 base pair differences were combined in a precluster step, following which chimeras were identified and removed using UCHIME [57]. Sequences were then classified using the naive Bayesian classifier [58] and Ribosomal Database Project (RDP) taxonomy database (v16) [59]. Phylotype classification was used to identify sequences to the phylogenetic level of genus, and a taxonomy summary table produced.

Sixty-two genera were identified across all samples. Taxa that were identified with a higher abundance in water controls than in samples (based on a fold calculation) were removed as contaminants, with the exception of *Pseudomonas*. This taxon was detected at a similar level of low abundance in both samples and water control, but as we have previously detected *Pseudomonas* in CVL samples using mass spectrometry, we elected to include this genus [10]. Replicates for each biological sample were pooled, and for the purposes of visualization, the top 25% most abundant taxa overall are shown, while remaining lower abundance taxa have been binned to “other.”

#### Swedish cis vaginal 16S rRNA gene analysis

DNA extraction, targeted amplification, and sequencing of the V3–V4 regions of the 16S rRNA gene from vaginal mucosal samples were performed as described previously [60].

#### TW neovaginal and rectal mucosal 16S rRNA gene analysis

DNA extraction, targeted amplification, and sequencing of the V4 region of the 16S rRNA gene were performed as previously described [61]. Sequences were demultiplexed with Golay error correction using QIIME 1.9.1. Divisive amplicon denoising algorithm version 2 (DADA2) was used for error correction, exact sequence inference, and chimera removal [62]. Taxonomic assignment was performed using the RDP classifier with trainset 16 (10.5281/zenodo.801827).

#### Statistical analysis

Differential protein expression was performed using non-parametric Mann-Whitney *U* tests. Power calculations were performed in G*Power (v3.1.9.2). We were able to detect host proteome differences of 2.7-fold between samples taken from cis vaginas (*n* = 30) and neovaginas (*n* = 5) while retaining 80% power, assuming a proteome variance of 100% [60], an adjusted alpha = 0.0001. Alpha (Shannon’s *H* index) and beta diversity (Bray-Curtis dissimilarity distances) calculations as well as permutational multivariate analysis of variance (perMANOVA) calculations were performed in R (v3.5.0) using the vegan.R (v2.5-3) package. Principal coordinate analysis was conducted in R using ape.R (v5.2) package. The phyloseq.R (v1.16) package was also used for the analysis of 16S microbiome profiling data. Principal component analysis was conducted in MatLab and EigenVector software. Graphs and statistical analysis for bacterial protein function were generated using the functional microbiome analysis pipeline (LOGAN) [63]. Hierarchical clustering was conducted in R using NMF.R (v0.21.0). Pearson distance metrics and complete linkage were the parameters specified. Human protein functional analysis was conducted using over-representation analysis via ConsensusPathDB (Max Planck Institute for Molecular Genetics). Pathway-based sets (INOH, Reactome, KEGG) and gene ontology biological processes level 5 categories were selected. *p* values were calculated using hypergeometric tests.

## Supplementary information

**Additional file 1: Supplemental Figure 1.** Microbial profiles of neovaginal secretions from transgender women. A) Microbial profiles as measured via 16S rRNA gene sequencing. B) Microbial profiles as measured via metaproteomics. NV6 had the penile inversion/scrotal graft surgical method with sigmoid colon graft extension, while the other neovaginas (NV1, NV2, NV4, and NV7) were penile inversion/scrotal graft method only.

**Additional file 2: Supplemental Figure 2.** Microbial profiles of vaginal secretions from cis women. A) Microbial profiles of Swedish cisgender women (CW) as measured via 16S rRNA gene sequencing. B) Microbial profiles of Swedish CW as measured via metaproteomics. C) Microbial profiles of Canadian CW as measured via 16S rRNA gene sequencing. D) Microbial profiles of Canadian CW as measured via metaproteomics.

**Additional file 3: Supplemental Figure 3.** Microbial profiles of rectal secretions from transgender women. A) Microbial profiles as measured via 16S rRNA gene sequencing. B) Microbial profiles as measured via metaproteomics.

**Additional file 4: Supplemental Figure 4.** Matched neovaginal and rectal samples show limited bacterial composition similarity. A) Bacterial composition profiles measured via metaprotemics. Microbial abundance was calculated by summing normalized total spectral counts for all proteins associated with each genus. B) Prinicipal coordinate analysis (PCoA) plots of Bray-Curtis distance of microbial composition. The composition was driven by sample type and *Roseburia* levels. Each dot represents one individual sample. (NV=neovaginal, R=rectal).

**Additional file 5: Supplemental Figure 5.** The functional profiles of cis vaginal and neovaginal samples based on bacterial proteins measured by mass spectrometry and functions assigned through the KEGG pathways database.

**Additional file 6: Supplemental Figure 6.** Protein set enrichment analysis identifies overlapping signatures measured in the neovagina of transgender women and in cervices of cisgender women with elevated activated CD4+ T cell levels. A) Pre-ranked proteins are plotted based on their enrichment score. Each dot on the plot represents overlapping proteins known to decrease in individuals with elevated cervical CD4+CD38+HLADR+ T cell levels as well as those known to decrease in neovagina relative to cis vagina. B) The rank metric score represents each protein’s correlation with the neovaginal phenotype. Proteins highlighted in blue represent those that account for the core enrichment, proteins that contribute most to the enrichment, observed.

**Additional file 7: Supplemental Table 1.** Percent coverage and mean normalized bacterial spectral counts of KEGG ko level functions from neovaginal and cis vaginal compartments. **Supplemental Table 2.** Proteins differentially abundant between neovaginas and cis vaginas. **Supplemental Table 3.** Enriched host pathways positively associated with the neovaginal compartment compared to the cis vaginal compartment. **Supplemental Table 4.** Enriched host pathways negatively associated with the neovaginal compartment compared to the cis vaginal compartment. **Supplemental Table 5.** Human protein correlates of bacterial diversity as measured by Shannon's H index. **Supplemental Table 6.** Enriched host pathways positively associated with bacterial diversity as measured by Shannon's H index. **Supplemental Table 7.** Enriched host pathways negatively associated with bacterial diversity as measured by Shannon's H index. **Supplemental Table 8.** Immune cell protein set signatures that overlap with signatures enriched in the neovagina. **Supplemental Table 9.** Taxa included in the curated database and each taxa's protein detection from initial TREMBL bacteria database search.

## Data Availability

16S rRNA gene sequence files and metadata for the TW and CW samples used in this study have been deposited in Figshare (https://figshare.com/articles/TW_neovaginal_rectal_buccal_seq/11690382; https://figshare.com/articles/CW_16S/11710227). Data sets including unrarefied OTU tables from 16S rRNA gene sequence data, metadata, protein spectral count data, and R scripts used in this study are available in GitHub (https://github.com/kmbirse/Birse_etal_Neovaginal-Microbiome).

## Abbreviations

BV: Bacterial vaginosis
C6: Complement component 6
CAMP: Cathelicidin antimicrobial peptide
CFH: Complement factor H
CRISP3: Cysteine-rich secretory protein 3
CST: Community state type
CW: Cisgender women
FDR: False discovery rate
GRS: Gender reassignment surgery
KEGG: Kyoto Encyclopedia of Genes and Genomes
KOALA: KEGG Orthology And Links Annotation
LC: Liquid chromatography
LD: *Lactobacillus* dominant
MS: Mass spectrometer
NES: Normalized enrichment score
nLD: Non-*Lactobacillus* dominant
PCoA: Principal coordinate analysis
perMANOVA: Permutational multivariate analysis of variance
QSOX1: Sulfhydryl oxidase 1
STI: Sexually transmitted infection
TLR: Toll-like receptor
TREMBL: Translated European Molecular Biology Laboratory Nucleotide Sequence Database; genome mapping software
TW: Transgender women
TXN: Thioredoxin
