# Supplementary figures and images for: The neovaginal microbiome of transgender women post-gender reassignment surgery
Source: Microbiome. 2020 May 5;8:61. doi: 10.1186/s40168-020-00804-1 (PMC7201977; doi:10.1186/s40168-020-00804-1)

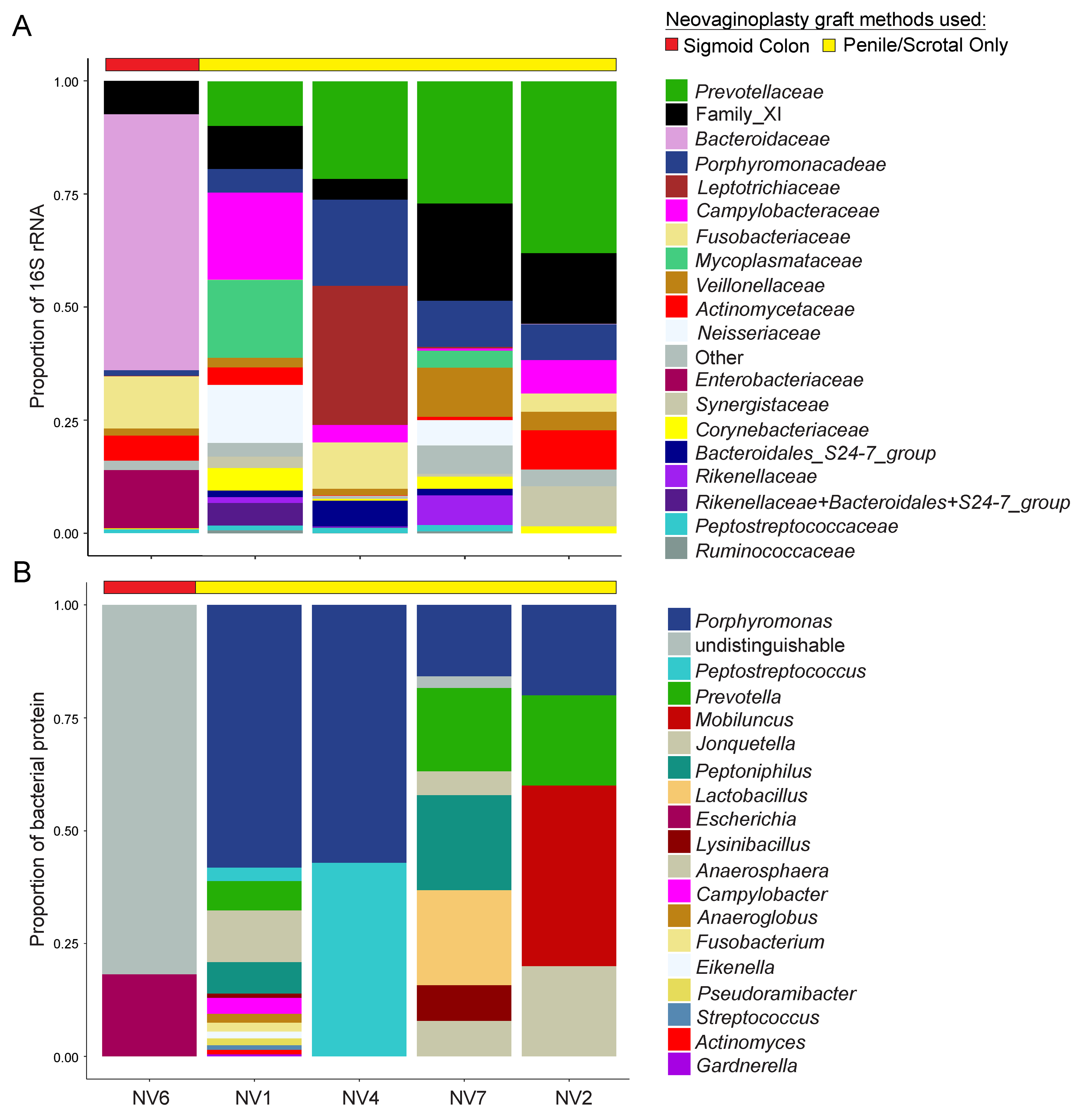

Supplement: Supplementary file 2 — Additional file 1: Supplemental Figure 1. Microbial profiles of neovaginal secretions from transgender women. A) Microbial profiles as measured via 16S rRNA gene sequencing. B) Microbial profiles as measured via metaproteomics. NV6 had the penile inversion/scrotal graft surgical method with sigmoid colon graft extension, while the other neovaginas (NV1, NV2, NV4, and NV7) were penile inversion/scrotal graft method only. [file 40168_2020_804_MOESM1_ESM.png]

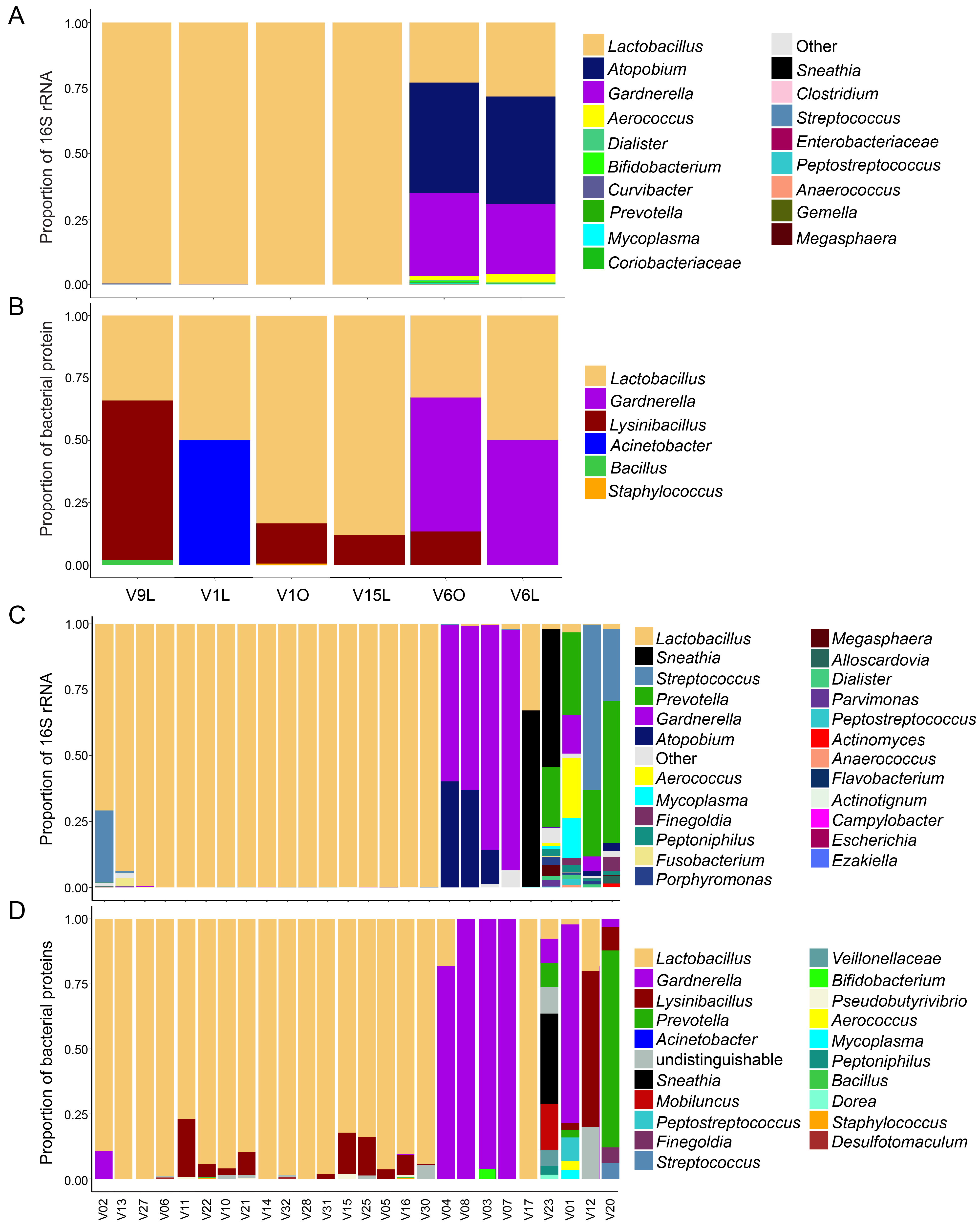

Supplement: Supplementary file 3 — Additional file 2: Supplemental Figure 2. Microbial profiles of vaginal secretions from cis women. A) Microbial profiles of Swedish cisgender women (CW) as measured via 16S rRNA gene sequencing. B) Microbial profiles of Swedish CW as measured via metaproteomics. C) Microbial profiles of Canadian CW as measured via 16S rRNA gene sequencing. D) Microbial profiles of Canadian CW as measured via metaproteomics. [file 40168_2020_804_MOESM2_ESM.png]

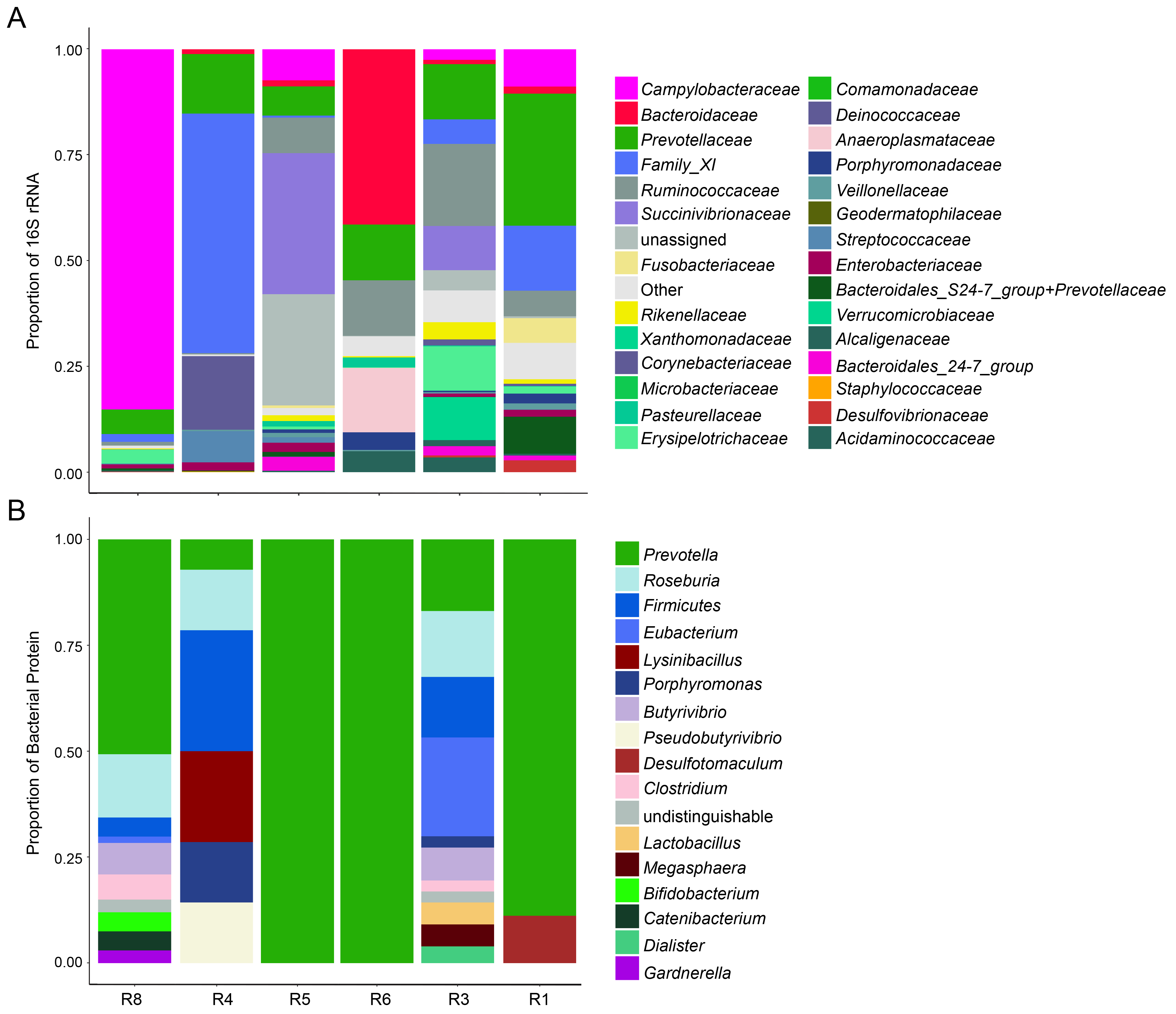

Supplement: Supplementary file 4 — Additional file 3: Supplemental Figure 3. Microbial profiles of rectal secretions from transgender women. A) Microbial profiles as measured via 16S rRNA gene sequencing. B) Microbial profiles as measured via metaproteomics. [file 40168_2020_804_MOESM3_ESM.png]

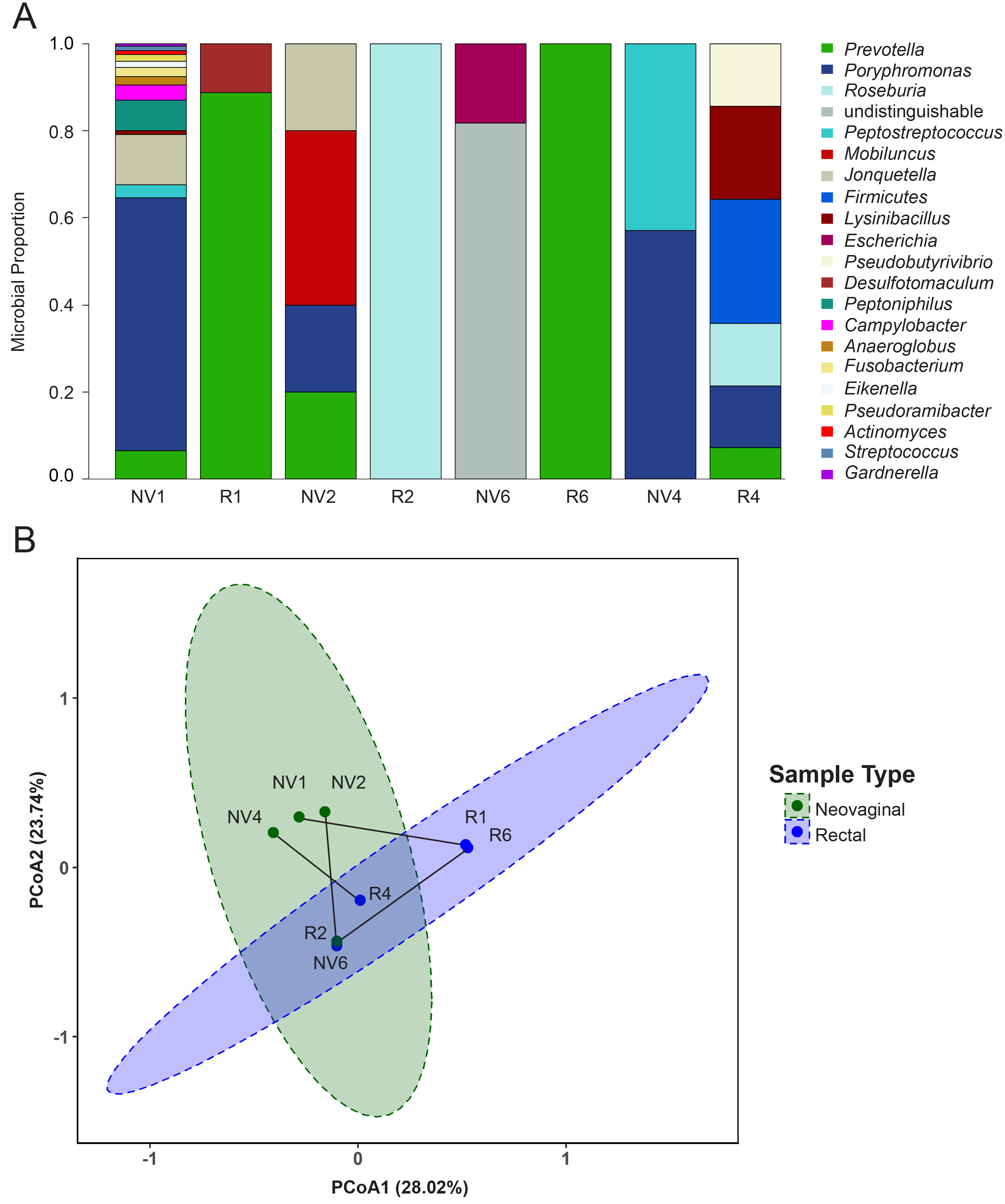

Supplement: Supplementary file 5 — Additional file 4: Supplemental Figure 4. Matched neovaginal and rectal samples show limited bacterial composition similarity. A) Bacterial composition profiles measured via metaprotemics. Microbial abundance was calculated by summing normalized total spectral counts for all proteins associated with each genus. B) Prinicipal coordinate analysis (PCoA) plots of Bray-Curtis distance of microbial composition. The composition was driven by sample type and Roseburia levels. Each dot represents one individual sample. (NV=neovaginal, R=rectal). [file 40168_2020_804_MOESM4_ESM.png]

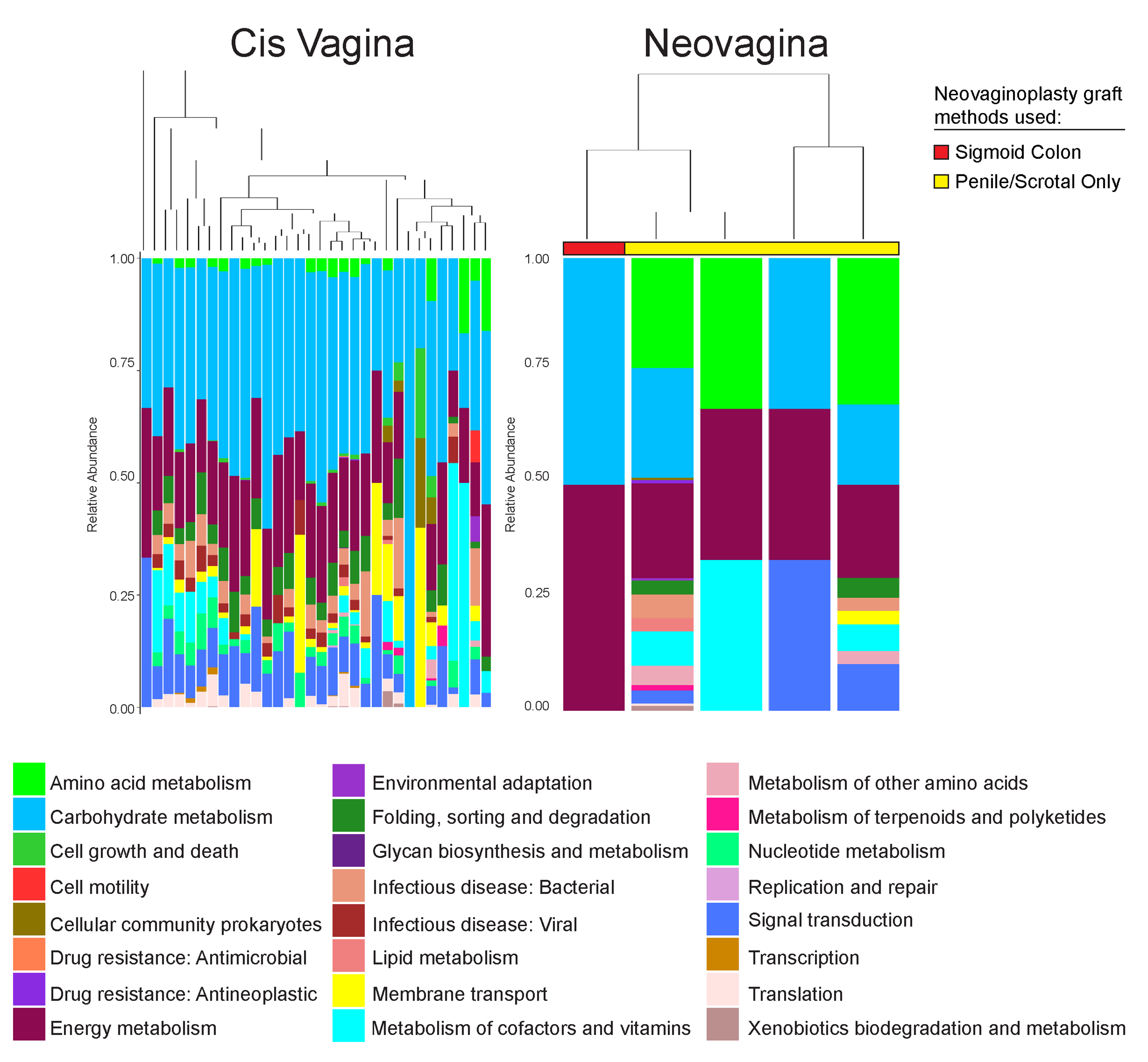

Supplement: Supplementary file 6 — Additional file 5: Supplemental Figure 5. The functional profiles of cis vaginal and neovaginal samples based on bacterial proteins measured by mass spectrometry and functions assigned through the KEGG pathways database. [file 40168_2020_804_MOESM5_ESM.png]

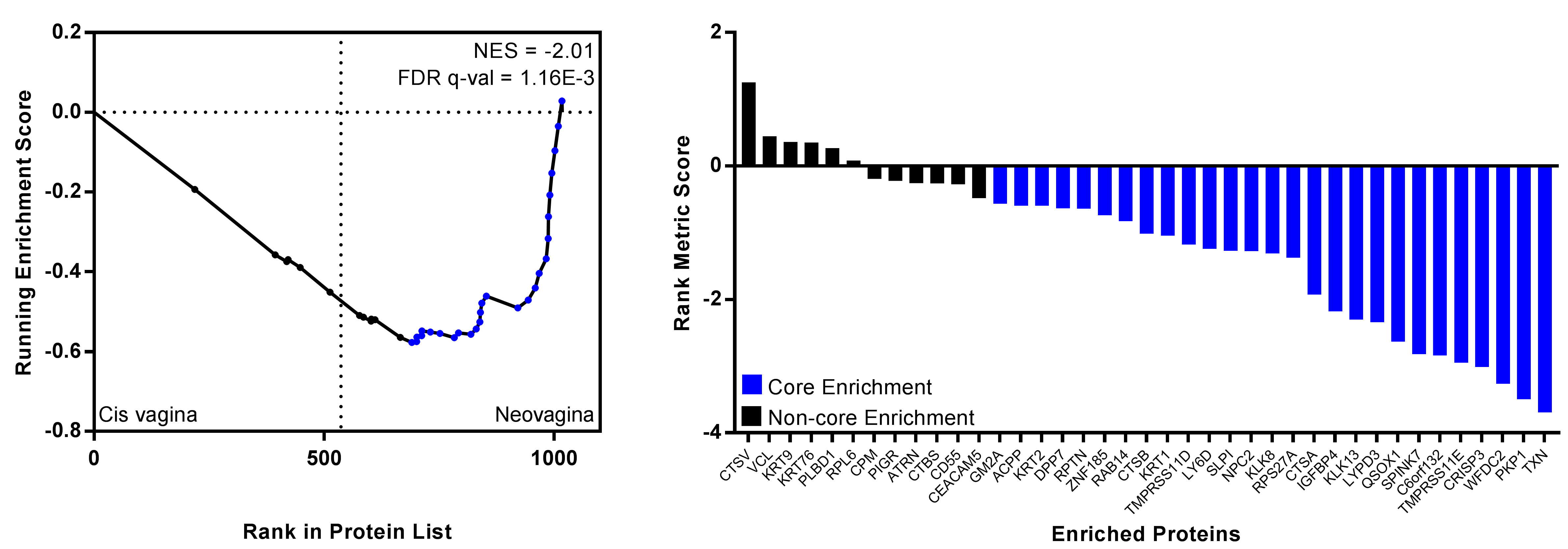

Supplement: Supplementary file 7 — Additional file 6: Supplemental Figure 6. Protein set enrichment analysis identifies overlapping signatures measured in the neovagina of transgender women and in cervices of cisgender women with elevated activated CD4+ T cell levels. A) Pre-ranked proteins are plotted based on their enrichment score. Each dot on the plot represents overlapping proteins known to decrease in individuals with elevated cervical CD4+CD38+HLADR+ T cell levels as well as those known to decrease in neovagina relative to cis vagina. B) The rank metric score represents each protein’s correlation with the neovaginal phenotype. Proteins highlighted in blue represent those that account for the core enrichment, proteins that contribute most to the enrichment, observed. [file 40168_2020_804_MOESM6_ESM.png]
